# Olfaction as a Marker for Dystonia: Background, Current State and Directions

**DOI:** 10.3390/brainsci10100727

**Published:** 2020-10-13

**Authors:** Thorsten Herr, Julie Gamain, Robert Fleischmann, Bernhard Lehnert, Marcus Vollmer, Carsten Willert, Birgitt Veit, Andrea Stenner, Jan-Uwe Mueller, Barbara Caspers, Martin Kronenbuerger

**Affiliations:** 1 Department of Neurology, University Medicine Greifswald, Fleischmannstraße 8, 17475 Greifswald, Germany; thorsten.herr@web.de (T.H.); gamain.julie@yahoo.com (J.G.); Robert.Fleischmann@med.uni-greifswald.de (R.F.); 2 Department of Otorhinolaryngology, University Medicine Greifswald, Fleischmannstraße 8, 17475 Greifswald, Germany; bernhard.lehnert@med.uni-greifswald.de; 3 Institute of Bioinformatics, University Medicine Greifswald, Felix-Hausdorff-Str. 8, 17475 Greifswald, Germany; marcus.vollmer@uni-greifswald.de; 4 Neurology Group Practice, Bleistraße 13, 18439 Stralsund, Germany; c-willert@telemed.de; 5 Neurology Group Practice, An der Marienkirche 2, 17033 Neubrandenburg, Germany; b.veit@medizin-nb.de; 6 Outpatient Department of Neurology, Movement Disorders Competence Center, Paracelsus Hospital Zwickau, Werdauer Straße 68, 08060 Zwickau, Germany; dr.andrea.stenner@pk-mx.de; 7 Department of Neurosurgery, University Medicine Greifswald, Fleischmannstraße 8, 17475 Greifswald, Germany; jan-uwe.mueller@med.uni-greifswald.de; 8 Department of Behavioural Ecology, University of Bielefeld, Konsequenz 45, 33615 Bielefeld, Germany; barbara.caspers@uni-bielefeld.de; 9 Department of Neurology, Johns Hopkins University, 600 N. Wolfe Street, Meyer 6-181B, Baltimore, MD 21287, USA; 10 Department of Neurology, Medical School OWL, University of Bielefeld, Campus Lippe, Rintelner Straße 85, 32657 Lemgo, Germany

**Keywords:** chemical senses, taste, non-motor manifestation, basal ganglia, cerebellum, sensorimotor cortex, network, social interactions

## Abstract

Dystonia is a heterogeneous group of hyperkinetic movement disorders. The unifying descriptor of dystonia is the motor manifestation, characterized by continuous or intermittent contractions of muscles that cause abnormal movements and postures. Additionally, there are psychiatric, cognitive, and sensory alterations that are possible or putative non-motor manifestations of dystonia. The pathophysiology of dystonia is incompletely understood. A better understanding of dystonia pathophysiology is highly relevant in the amelioration of significant disability associated with motor and non-motor manifestations of dystonia. Recently, diminished olfaction was found to be a potential non-motor manifestation that may worsen the situation of subjects with dystonia. Yet, this finding may also shed light into dystonia pathophysiology and yield novel treatment options. This article aims to provide background information on dystonia and the current understanding of its pathophysiology, including the key structures involved, namely, the basal ganglia, cerebellum, and sensorimotor cortex. Additionally, involvement of these structures in the chemical senses are reviewed to provide an overview on how olfactory (and gustatory) deficits may occur in dystonia. Finally, we describe the present findings on altered chemical senses in dystonia and discuss directions of research on olfactory dysfunction as a marker in dystonia.

## 1. Introduction

In clinical neuroscience, olfactory decline is typically associated with sinunasal disease, head trauma, infections of the upper respiratory tract, dementing illnesses such as Alzheimer’s disease, and movement disorders such as Parkinson’s disease [1,2,3]. Just like Parkinson’s disease, dystonia is a common movement disorder [4]. Until recently, dystonia had not been associated with diminished sense of smell (and taste).

Dystonia is characterized by sustained or intermittent muscle contractions that cause abnormal movements and postures [5]. The group of neurological syndromes in which dystonia occurs is heterogeneous and thus classified according to clinical characteristics (axis 1) and etiology (axis 2) [5]. The most common form of dystonia encountered in adult neurology is focal dystonia [4]. Within the group of focal dystonia, cervical dystonia is the most prevalent type, being characterized by a sustained or jerky position of the neck [4,6]. The second and third most common types of focal dystonia are, respectively, blepharospasm (characterized by spasms of the eyelid [7]) and writer’s cramp (characterized by dystonia during writing [4]). Besides the motor manifestations, there are numerous non-motor alterations in the different forms of dystonia [8,9]. These include psychiatric alterations (such as depression and anxiety), mild cognitive deficits (such as deficits in executive functioning, verbal fluency, and memory), and changes in the sensory domain (such as pain, kinesthetic sensation, impaired somatosensory discrimination, and alleviating maneuvers) [8,9].

As a non-motor deficit, diminished sense of smell can be found in various movement disorders [10]. Mild impairment of the sense of smell was found in restless leg syndrome [11], degenerative ataxia [12,13,14,15,16], essential tremor [17,18,19,20], Huntington’s disease [21,22,23,24], Tourette syndrome [25], and atypical Parkinson syndromes [10,26]. Moreover, in the majority of cases of Parkinson’s disease, impairment of the sense of smell is severe [27,28,29]. Little is known about the diminished sense of taste in movement disorders. Parkinson’s disease is associated with an impaired sense of taste [30,31,32,33], but gustatory ability has not yet been systematically assessed in other movement disorders. This is surprising, since the sense of smell and the sense of taste work together in daily life [34,35]. Thus, comprehensive assessment of the chemical senses should cover both olfactory and gustatory ability.

Although largely overlooked, the chemical senses fulfill fundamental aspects important for quality of life, safety, and social interactions [34,35]. They contribute to quality of life, such as the joy of smelling a pleasant odor or the pleasure of tasting of such wonders as chocolate. Additionally, chemical senses can warn us of hazards, such as the smell of smoke from fire or the sour taste of spoiled milk. Moreover, olfaction impacts social and family relations, such as mother–child bonding [36] and intimate relationships [37,38]. Olfactory impairment can also have negative consequences. For example, people with a diminished sense of smell can have major impairments in their professional lives, particularly individuals who work in the gastronomy, the perfume industry, or the healthcare system [34,39,40]. Furthermore, subjects with olfactory impairment may have problems with hygiene because they are no longer able to detect their own body odor [39,40,41]. 

In addition to the role of the chemical senses in daily life, olfactory and gustatory abilities may contribute to understanding the diseases of the nervous system. The architecture of the olfactory system and the architecture of the gustatory system are well described, and the pattern of impairment in multi-component tests may reflect underlying pathology [1,42,43,44,45,46]. For instance, impairment in odor threshold tests and normal performance with tasks involving supra-threshold odor concentrations (such as odor identification and odor discrimination) point to an impairment of the peripheral olfactory system, such as that seen in sinunasal disease [1,47]. In contrast, normal performance in tests for odor threshold, but diminished performance in tests for odor identification and odor discrimination, is typically seen in diseases of the central nervous system, such as Parkinson’s disease [1,47]. There are exceptions, however. For instance, in cerebellar disease, an impairment of odor threshold and odor identification can be found [48,49]. 

Although it is a common movement disorder, the pathophysiology of dystonia is incompletely understood [50,51,52,53,54,55,56]. A better understanding of dystonia pathophysiology is highly relevant because motor manifestations and non-motor alterations in dystonia cause significant disability [9,57,58,59,60]. For instance, more than 70% of the subjects with focal dystonia suffer from depression, anxiety, and pain [8,9]. Additionally, about 60% of the subjects with cervical dystonia have decreased vocational productivity and about 40% lost their jobs because of dystonia [57]. Diminished olfaction may worsen the situation of subjects with dystonia because of the negative effects of impaired sense of smell on quality of life, safety, and social interactions. Moreover, current treatment options for dystonia, such as injections with botulinum toxin [61] or deep brain stimulation [62], are symptomatic and improve only certain subtypes of dystonia [63,64,65,66,67]. A better understanding of dystonia may lead to novel treatment approaches for subjects with dystonia.

## 2. Current Understanding of Dystonia Pathophysiology

Several methods of research have been applied to unfold the pathophysiology of dystonia [50,51,52,53,54,55,56,68]. The most relevant tools for this purpose are likely neuroimaging, neurophysiological studies, and clinical studies (in particular, tremor), as they allow for the study of human subjects who actually have dystonia. Findings are reviewed in brief.

Standard neuroimaging such as computed tomography (CT) and magnetic resonance imaging (MRI) are typically normal in subjects with primary dystonia [69,70,71,72]. In rare cases, however, focal lesions can produce a clinical picture nearly identical to idiopathic dystonia [73,74,75]. Lesions in secondary dystonia were most commonly located in a network including the basal ganglia, cerebellum, and sensorimotor cortex [73,75,76,77,78]. Findings of advanced neuroimaging techniques (such as diffusion tensor imaging, voxel-based morphometry, resting state functional MRI, and positron emission tomography (PET)) revealed alterations within these structures and thus are consistent with findings from clinicopathological studies [52,55,70,71,75,76,77,78,79]. 

Besides neuroimaging, neurophysiology is a powerful tool to aid in the understanding of dystonia pathophysiology [54,80,81,82]. Three principal abnormalities were identified: (i) loss of lateral inhibition, (ii) alterations in the sensory domain, and (iii) maladaptive plasticity. Findings from several studies that used different neurophysiological methods (such as electromyography and transcranial magnetic stimulation) indicate that the basal ganglia, cerebellum, and sensorimotor cortex are involved in these abnormalities [80,83,84,85,86,87,88,89,90,91,92,93,94,95,96,97,98,99,100,101,102,103,104,105,106,107,108,109,110,111]. 

Furthermore, several studies assessed clinical alterations in subjects with idiopathic dystonia. Subtle alterations were found with saccadic adaptation, posture, and gait in subjects with dystonia [112,113,114,115,116]. Findings of these studies point to subtle cerebellar alterations in dystonia. In addition to dystonia, tremor has been found to be the most obvious and common clinical symptom in addition to dystonia [5,117]. In studies of tremor in adult-onset focal dystonia, the majority of the subjects (51–65%) with cervical dystonia [6,118,119] and about 20% of the subjects with blepharospasm had action tremor [118]. Moreover, a recent review suggests that up to 12% of the subjects with primary dystonia have rest tremor, with most of them having multifocal and segmental dystonia [120]. Although the two types of tremor are different with regard to when they are present (rest vs. action), both are related to altered activity within a network including the basal ganglia, cerebellum, and sensorimotor cortex, as suggested by functional imaging studies [120,121,122]. 

In summary, findings from studies using different methods of research by different, independent research groups suggest that dystonia pathophysiology is related to dysfunction of the basal ganglia, cerebellum, and sensorimotor cortex, as well as the interactions between these structures.

## 3. Involvement of the Basal Ganglia, Cerebellum, and Sensorimotor Cortex in Olfaction

The architecture of the olfactory system and architecture of the gustatory system are well described [42,43,123,124]. The anatomy of both sensory systems is briefly reviewed. 

**Figure 1:**
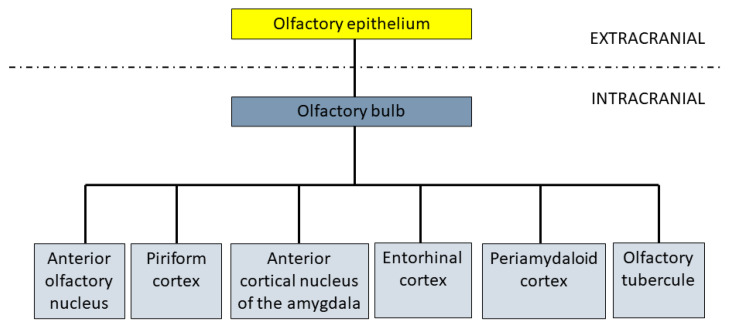
Schematic illustration of key olfactory pathways.

The olfactory epithelium is located at the upper aspect of the nasal cavities [43,44,125]. Odorant molecules activate the olfactory receptor neurons in the olfactory epithelium [43,44,125]. These synapse with the mitral cells of the olfactory bulbs [43,44]. Projections of the olfactory bulb via the olfactory tracts broadly define the olfactory cortex [43,44,45]. This includes the anterior olfactory nucleus, piriform cortex, anterior cortical amygdaloid nucleus, periamygdaloid cortex, and lateral entorhinal cortex [43,44,45] (Figure 1).

These structures in turn project to several other brain regions such as the orbitofrontal cortex, hippocampus, hypothalamus, thalamus, cingulate gyrus, and insular cortex, as well as the basal ganglia and cerebellum [43,44]. 

As the olfactory and the gustatory system interact in daily life, both systems need to be considered to fully understand the chemical senses [47,126]. The architecture of the gustatory system is very different from that of the olfactory system [42,43,127]. While parts of the olfactory system are considered an evagination of the brain, parts of the gustatory system are derivatives of the skin [42,43]. At the peripheral aspect are the taste receptors cells, which are mainly located at the dorsal and lateral aspects of the tongue and are activated by flavor molecules [42,43,128]. Theses taste receptor cells are connected with the cranial nerves VII, IX, and X [42,43,129]. The axons of these cranial nerves, which are involved in taste, reach the brain stem and synapse with neurons of the solitary tract [42,43,127,130]. The axons of these neurons in turn ascend either through the central tegmental tract to the ipsilateral ventral posterior medial nucleus of the thalamus, or with the medial lemniscus to the contralateral thalamic counterpart [127,131,132]. From these thalamic nuclei the gustatory signals reach the insula cortex and the sensorimotor cortex [132,133,134,135,136,137]. 

The involvement of the basal ganglia, cerebellum, and sensorimotor cortex in the chemical senses, as mentioned in standard resources, appears surprising at first, since these anatomical structures are traditionally considered to be concerned with sensorimotor control. However, an abundance of evidence from tracing studies, functional imaging studies, and clinicopathological studies revealed that the anatomical structures named above are interconnected with structures of the olfactory and gustatory system. 

Several tracing studies in rats and non-human primates have shown that the striatum is both directly and indirectly connected with the olfactory cortex [138,139,140,141,142,143,144]. Likewise, the cerebellum receives input from the olfactory cortex via the ventral tegmental area [145,146]. In addition to the tracing studies in animals, functional imaging studies in humans showed an involvement of the basal ganglia and the cerebellum in olfaction. Both structures were activated with olfactory tasks in PET studies in healthy human subjects [63,147,148]. This activation was also found in functional magnetic resonance imaging (fMRI) studies [149,150]. Furthermore, cerebellar involvement in olfaction is also supported by clinicopathological studies. For instance, subjects with degenerative disease of the cerebellum were found to have olfactory deficits [14,16]. Additionally, focal cerebellar lesions, such as vascular lesions or cerebellar tumors, cause olfactory decline, as assessed with controlled clinical and neurophysiological studies [48,49]. Moreover, the cerebellum and its efferent projection (namely, the ventrolateral thalamus and sensorimotor cortex) are important parts of an olfacto-motor loop [48]. Within this circuit, the cerebellum as a structure for sensorimotor control regulates sniff volume inversely proportional to odor concentration in order to optimize the sampling of sensory information [48,49,151,152]. 

In contrast to the cerebellum, which probably does not receive gustatory input, several studies revealed that the basal ganglia are involved in the sense of taste. For example, parts of the solitary tract of the brain stem project to the amygdala [153,154], which in turn project to the striatum, as revealed by tracing studies [142,143]. Likewise, the (gustatory) insular cortex projects to the striatum [155]. Furthermore, the involvement of the basal ganglia in the sense of taste is supported by functional imaging. A high-field MRI study showed activation of the striatum with gustatory tasks in healthy human subjects [156]. 

The sensorimotor cortex represents the somatosensory input from the limbs, face, and tongue [131]. Additionally, it receives afferent projections from the ventral posterior medial nucleus of the thalamus and is therefore also involved in processing information related to the sense of taste [42,131]. This finding is consistent with an imaging study [136]. Salt and water (both considered independent taste modalities) that were applied to one hemitongue resulted in an activation of the insular cortex and the inferior part of the postcentral gyrus in healthy human subjects [157]. Additionally, a clinicopathological study that was assessing subjects with chronic cerebral lesions reiterated that the insular cortex and the somatosensory cortex are important for processing information related to the sense of taste [157].

Thus, several lines of evidence have shown that the basal ganglia, cerebellum, and sensorimotor cortex are involved in both olfaction and the sense of taste.

## 4. Olfactory Deficits in Dystonia: The Current State

On the basis of the anatomical overlap of the involvement of the basal ganglia, cerebellum, and sensorimotor cortex in the pathophysiology of dystonia and the sense of smell, olfactory alterations may be expected in subjects with dystonia. Over the last 11 years, four studies have been published that showed diminished olfaction in subjects with primary dystonia (please see Table 1 for details). 

The first study on olfaction in dystonia was a study of olfaction in subjects with “scans without evidence of dopaminergic deficit” (SWEDDs) [158]. For comparison with SWEDDs subjects, the researchers studied healthy controls and subjects with different forms of idiopathic adult onset dystonia—12 of them had cervical dystonia, 2 had dystonia with tremor of the arm, 1 had writer’s cramp, and there was 1 with blepharospasm. For olfactory assessment, the University of Pennsylvania Smell Inventory Test (UPSIT) [162] was applied. The subjects with dystonia that were assessed had a slightly diminished UPSIT score compared to healthy controls, but the difference was not statistically different (see Table 1). The lack of statistical difference could be related to the small number of dystonia subjects assessed, the number of female participants, and the difference in age of the study groups. 

Another study focused on the genetic assessment of subjects with a *GNAL* mutation [159]. *GNAL* encodes the guanine nucleotide-binding protein, alpha activating activity polypeptide, olfactory type (Ga(olf), Golfalpha). It is involved in signal transduction in the olfactory system as well as in the basal ganglia and the cerebellum [159,165,166]. Although this type of dystonia is rare, *GNAL* mutation is of interest in terms of olfaction in dystonia. As part of the clinical protocol, olfactory testing was performed in subjects with the *GNAL* mutation [159]. Using the UPSIT, the researchers assessed 14 healthy controls (all were non-carriers), 8 asymptomatic gene carriers, and 7 symptomatic dystonia subjects (most of them had cervical dystonia and segmental dystonia) from four different, biologically unrelated families. The UPSIT score of the symptomatic dystonia subjects was found to be lower than the UPSIT score of the asymptomatic gene carriers and the healthy control subjects, but the differences were not statistically different. A subgroup analysis in only one family however revealed statistically significant differences in the UPSIT scores between subjects with the *GNAL* mutation and healthy controls (Table 1). 

The first study, which primarily focused on olfaction in idiopathic dystonia, assessed 58 subjects with cervical dystonia and compared findings to the data of two groups of healthy controls from a database (Table 1) [160]. For olfactory assessment, so-called Sniffin Sticks were used [163]. Sniffin Sticks allow for the assessment of odor threshold and odor discrimination in addition to odor identification. The sum of the results of the three sub-scores is combined to the threshold, discrimination and identification (TDI) score. A TDI score of 0 represents a poor result while a score of 48 is an excellent result. This score is important because a result below 30 indicates hyposmia and denotes an olfactory impairment that impacts daily life [167]. Compared to the data of the two groups of healthy controls from a database, subjects with cervical dystonia had a statistically significant lower score for odor threshold and odor identification, while performance in the odor discrimination test was not statistically different. Moreover, more subjects with cervical dystonia were found to have hyposmia compared to healthy controls in the two control groups (cervical dystonia subjects: 50%; healthy controls group 1: 20.7%; healthy controls group 2: 22.4%; *p* = 0.001). Clinical parameters, such as the degree of cervical dystonia, did not predict olfactory decline. Non-motor manifestations of dystonia, such as cognitive and psychiatric alterations, which potentially impact the performance of olfactory tests [161,164,168,169,170,171,172,173,174,175,176,177,178,179], were not assessed as co-factors in this study. 

To comprehensively assess the chemical sense in dystonia, a more recent study appraised the sense of smell, as well as the sense of taste in subjects with cervical dystonia, which was compared to carefully matched healthy controls (Table 1) [161]. Moreover, cognitive and psychiatric alterations as non-motor manifestations of dystonia were assessed by the use of an extensive test battery. For the best possible comparison of the two groups and to exclude other causes that could impact the chemical senses as suggested by a consensus panel [47], the researchers applied an extensive list of exclusion criteria [161]. Dystonia subjects on treatment with botulinum toxin were assessed 3 months after their last injections, when the effects of botulinum toxin treatment had wasted. All study participants were assessed by following the same rigorous study protocol. The Sniffin Sticks were used for the assessment of the odor threshold, odor discrimination, and odor identification [163]. For the assessment of the sense of taste, the study employed Taste Strips [164]. These are used to assess four taste qualities (sweet, sour, salty, and bitter) in four different concentrations. The score of the Taste Strips ranged from 0 (poor result) to 16 (excellent result). A taste score below 9 was defined as a hypogeusia, which denotes gustatory impairment with functional significance [172]. Consistent with findings of an independent study on olfaction in subjects with cervical dystonia [160], this study found that the odor threshold was lower in subjects with dystonia than in healthy control subjects. Moreover, odor identification was lower in subjects with cervical dystonia than in healthy controls, while odor discrimination was not altered in the dystonia subjects. Additionally, 52.2% of the cervical dystonia subjects had hyposmia, while 22.5% of the healthy control subjects had a low score (*p* = 0.003). Furthermore, the combined taste score that was assessed with the Taste Strips was lower in subjects with cervical dystonia than in healthy controls. Consistent with findings from epidemiological studies [35,172,173], the analyses of co-factors revealed that age was a predictor of olfactory decline. Psychiatric and cognitive alterations of the dystonia subjects did not correlate with the performance of the chemical senses. Interestingly, pain in dystonia was a predictor for low odor threshold. Most importantly, motor deficits did not predict low performance on the tests of the chemical senses. Therefore, this study suggests that olfactory and gustatory deficits in dystonia are neither secondary to motor deficits nor the product of psychiatric and cognitive alterations.

There are few reports on olfaction in subjects with combined dystonia, such as Lubag syndrome [174,175], spinocerebellar ataxia type 3 [176], and neuronopathic Gaucher [177]. Mild to moderate olfactory decline was found in these subjects [174,175,176,177]. The results are not reviewed in detail here because the pathophysiology of these combined dystonia syndromes may be different from primary dystonia.

In sum, findings of past studies suggest that olfactory (and gustatory) decline can be found in dystonia. Specifically, detailed studies revealed that subjects with cervical dystonia had an impairment of odor threshold and odor identification, which were unrelated to motor and non-motor deficits. Additionally, in about 50% of the cases, cervical dystonia comes with hyposmia, an olfactory impairment which most likely impacts daily life. Therefore, olfactory decline is common and may represent an independent non-motor manifestation of cervical dystonia. As different structures are involved in the pathophysiology of dystonia and the processing of olfactory information, it is difficult to pin-point findings to a single site of pathology. Yet, findings of altered chemical senses may support the idea that dystonia is related to alterations in a network including the basal ganglia, cerebellum, and sensorimotor cortex. 

## 5. Olfaction as a Potential Marker for Dystonia: Further Directions

A (disease) marker is either a substance, a measurable parameter, or a physical sign of a disease [178,179,180,181]. There are two types of markers: trait markers and state markers. A trait marker can be used to diagnose a disease in a subject or to identify a subject at risk. Typically, a trait marker persists over time. In contrast to a trait marker, a state marker correlates with the degree of a disease. Typically, a state marker disappears or diminishes after the remission or treatment of a disease. 

Olfactory decline in subjects with dystonia is a fairly recent discovery. Additionally, only a handful of studies have assessed a relatively limited number of dystonia subjects compared to healthy controls. It is possibly too early to define olfaction as a trait marker for dystonia. However, two independent studies revealed diminished odor threshold and diminished odor identification as the pattern of olfactory decline in subjects with cervical dystonia [160,161]. Findings are possibly related to the involvement of the basal ganglia, cerebellum, and sensorimotor cortex in both the pathophysiology of dystonia and the processing of olfactory information. Several types of dystonia share the involvement of the basal ganglia, cerebellum, and sensorimotor cortex in their pathophysiology. Therefore, olfactory decline may be found in other forms of focal dystonia such as blepharospasm or writer’s cramp. Functional imaging studies suggest that the degree of alterations of the basal ganglia, cerebellum, and sensorimotor cortex is found to vary according to the particular forms of the focal dystonias [182,183]. Further research may answer the question as to whether the pattern of olfactory decline differs in multicomponent olfactory tests in the different forms of focal dystonia. Moreover, it may be of interest if olfaction in subjects with inherited forms of dystonia differs from olfaction in subjects with idiopathic forms. Selected subjects with a mutation in *GNAL* were found to have deficits with odor identification [159]. Olfactory impairment in subjects with more prevalent forms of inherited dystonias such as TOR1A, THAP1, C1Z1, and ANO3 are unknown at present and may be assessed in future studies. It is of note that the presence of a genetic mutation in a movement disorder does not necessarily lead to olfactory decline. For example, in contrast to the sporadic form of Parkinson’s disease, subjects with PARK2, a common form of genetically determined Parkinson’s disease, do not have olfactory decline [27,184]. 

A state marker may be used to assess the response to a treatment or to predict the course of a disease. About 15% of the subjects with cervical dystonia and about 6% of the subjects with blepharospasm have a remission of dystonia [185]. Unfortunately, most of these subjects relapse within 4 to 5 years [185]. Furthermore, in some cases, dystonia spreads to other body parts [186]. The factors for remission, relapse, and spread in focal dystonia are not absolutely clear [185,186]. Longitudinal studies in subjects with different forms of dystonia may reveal if olfactory or gustatory decline is a risk factor for these phenomena.

Injections of botulinum toxin into muscles affected by dystonia is a well-established and very effective treatment [61,187,188,189]. To the best of our knowledge, effects of botulinum toxin on the chemical sense by comparing performance in tests of the chemical senses before and after injections of botulinum toxin have not been systematically assessed in subjects with dystonia. Findings of studies assessing a larger number of subjects with dystonia revealed that about one-third of the subjects with cervical dystonia and about 10% of the subjects with blepharospasm report dry mouth as a side effect of botulinum toxin treatment [61]. As saliva production in the mouth and mucus production in the nose are important for the chemical sense [190], decline of the sense of smell and taste may occur after botulinum treatment. On the other hand, studies using functional imaging found a tendency of improved altered activity in a network including the basal ganglia, cerebellum, and cortex after injections with botulinum toxin in subjects with dystonia [191,192,193,194,195,196]. Thus, in theory, botulinum treatment may also improve altered chemical sense in dystonia subjects. Therefore, an area of interest for future studies would be to see if botulinum treatment impacts (worsens or improves) the chemical senses in subjects with dystonia.

For selected subjects with dystonia, deep brain stimulation is an established therapy [62]. Typically, the basal ganglia are targeted in this type of dystonia treatment. Deep brain stimulation is also used to treat Parkinson’s disease and essential tremor. Several studies have reported the effects of deep brain stimulation on the chemical senses in subjects with Parkinson’s disease and essential tremor [20,35,197,198,199]. It is of interest as to whether deep brain stimulation for dystonia improves both the motor manifestation and the chemical senses in subjects with dystonia. Findings of such studies may add to the better understanding of dystonia as they could confirm that motor manifestations and impaired chemical senses in subjects with dystonia are caused by overlapping networks.

Finally, it is unclear at present as to how to therapeutically approach a decline of the chemical senses in dystonia. There is no established drug treatment for subjects with impairment of the chemical senses due to a central nervous system disease [47,200,201]. Olfactory training improved olfaction in subjects with Parkinson’s disease [124,202,203]. For olfactory training, subjects sniff on cotton balls impregnated with different odors. The subjects are instructed to exercise the sniffing for 15 seconds on each of the different cotton balls twice daily over a period of several weeks. As a non-invasive treatment option, further study may reveal if olfactory training might benefit subjects with dystonia and olfactory decline. 

## 6. Conclusions

Recent reports suggest that dystonia (especially cervical dystonia) may be associated with olfactory (and gustatory) decline. Olfactory deficits (and gustatory deficits) are possibly related to the overlap of a network involved in both the pathophysiology of dystonia and the processing of information related to the chemical senses. 

Current data suggest that about half of the subjects with cervical dystonia are affected by hyposmia. As the sense of smell is important for quality of life, safety, and social interaction, these findings demand further research of olfaction in dystonia. Future research may also address the question if olfaction is a trait marker (which helps to diagnose and differentiate the different forms of dystonia) or a state marker (which allows for the prediction of progression of dystonia or response to treatment). Moreover, treatment options for altered chemical senses in dystonia need to be identified. Olfactory training may be a candidate for this purpose. 

## Figures and Tables

**Table 1:** Summary of publications on olfaction in dystonia.

Study	n (%females)	Age in Years	Methods	Results	Conclusion(s)
Silveira et al. (2009) [158]	D: 16 (56) C: 136 (47)	D: 66.7 ± 6.8 C: 64.9 ± 8.8	UPSIT	D: 27.6 ± 7.5 C: 29.5 ± 5.3 *p* > 0.05	Difference not statistically different
Vemula et al. (2013) [159]	D: 6 * C: 5 *	not published	UPSIT	D: 25.5 ± 2.9 * C: 33.0 ± 1.1 * p < 0.026	subgroup has olfactory impairment
Marek et al. (2018) [160]	CD: 58 (66) C1: 58 (66) C2: 58 (66)	CD: 62.2 ± 11.8 C1: 62.2 ± 11.8 C2: 61.0 ± 12.8	Sniffin Sticks	TDI score CD: 28.9 ± 5.6 C1: 33.8 ± 5.6 C2: 32.2 ± 5.8 *p* < 0.001	impaired olfaction in cervical dystonia age correlated with low olfaction no correlation with motor manifestations
Herr et al. (2020) [161]	CD: 40 (58) C: 40 (58)	CD: 61.8 ± 10.9 C: 61.6 ± 12.2	Sniffin Sticks	TDI score CD: 29.5 ± 5.7 C: 33.9 ± 4.9 *p* < 0.001	impaired olfaction in cervical dystonia impaired taste in cervical dystonia age correlated with low olfaction no correlation with motor manifestations no correlation with non-motor alterations
			Taste Strips	composite score CD: 9.5 ± 2.2 C: 11.7 ± 2.7 *p* < 0.001	

Values are mean ± standard deviation; n number of subjects; D subjects with different forms of adult onset dystonia, C healthy control subjects, CD subjects with cervical dystonia; UPSIT University of Pennsylvania Smell Identification Test (0 poor; 40 best possible result) [162]; TDI score sum of odor threshold score, odor discrimination score and odor identification score of the Sniffin Sticks test [163]; Taste Strips [164] (0 poor; 16 best possible result). * Data of only one family assessed in the study by Vemula et al (2013) [159] are presented.
